# fMRI Supports the Sensorimotor Theory of Motor Resonance

**DOI:** 10.1371/journal.pone.0026859

**Published:** 2011-11-02

**Authors:** Claire Landmann, Sofia M. Landi, Scott T. Grafton, Valeria Della-Maggiore

**Affiliations:** 1 Department of Physiology and Biophysics, School of Medicine, University of Buenos Aires, Buenos Aires, Argentina; 2 Department of Psychology, University of California at Santa Barbara, Santa Barbara, California, United States of America; The University of Western Ontario, Canada

## Abstract

The neural mechanisms mediating the activation of the motor system during action observation, also known as motor resonance, are of major interest to the field of motor control. It has been proposed that motor resonance develops in infants through Hebbian plasticity of pathways connecting sensory and motor regions that fire simultaneously during imitation or self movement observation. A fundamental problem when testing this theory in adults is that most experimental paradigms involve actions that have been overpracticed throughout life. Here, we directly tested the sensorimotor theory of motor resonance by creating new visuomotor representations using abstract stimuli (motor symbols) and identifying the neural networks recruited through fMRI. We predicted that the network recruited during action observation and execution would overlap with that recruited during observation of new motor symbols. Our results indicate that a network consisting of premotor and posterior parietal cortex, the supplementary motor area, the inferior frontal gyrus and cerebellum was activated both by new motor symbols and by direct observation of the corresponding action. This tight spatial overlap underscores the importance of sensorimotor learning for motor resonance and further indicates that the physical characteristics of the perceived stimulus are irrelevant to the evoked response in the observer.

## Introduction

In primates, passive observation of actions performed by other individuals activates the motor system in a similar way to self-generated actions. Here, we will refer to this phenomenon as motor resonance. Elucidating the mechanism at the basis of motor resonance has been of major interest to the field of motor control since the identification of neurons with mirror activity in ventral premotor cortex (PMv) and inferior anterior intraparietal cortex (AIP) [1], [2]. A couple of theoretical accounts put forward by Heyes [3] and Keysers and Perret [4] postulate that motor resonance originates from sensorimotor associations (for a review on this topic see Heyes et al., 2010, [5]). According to these views, mirror properties develop in infants from Hebbian plasticity of pathways connecting sensory and motor regions firing simultaneously during imitation or self movement observation. Once the association is formed, perceiving the action would be sufficient to retrieve the sensorimotor network that became strengthened with practice.

A fundamental problem when testing the sensorimotor theory of motor resonance in adults is that most experimental paradigms used in the laboratory involve simple actions that have been overpracticed throughout life. On the other hand, new action sequences, no matter how difficult (e.g. dance), encompass motor primitives that already belong to the motor repertoire of the observer. Despite these limitations, one strategy to address this theory has been to experimentally alter the visuomotor mapping between the observed and the executed action through learning, and evaluate its impact on the motor system of the observer. Using this approach, Catmur and collaborators [6] showed that incongruent imitation consisting of executing an abduction movement with a different finger from the one observed, leads to changes in corticospinal excitability (CSE) consistent with the executed but not the observed action. fMRI examination further revealed that incongruent imitation reversed the activity of the action observation network, so that areas showing greater activity during observation of hand actions after congruent imitation responded more strongly to observation of foot actions after incongruent imitation [7]. These studies provide solid evidence indicating that motor resonance is not hard wired but can be modulated presumably as an outgrowth of extensive practice. Yet, they do not provide a direct demonstration that motor resonance originates from sensorimotor learning.

To overcome the limitations associated with using familiar action-perception pairings, we used abstract stimuli to create new visuomotor representations. Unlike objects or tools, abstract stimuli neither imply nor represent actions unless arbitrary paired with them. Visuomotor associations between abstract stimuli and actions have been long studied using the conditional motor learning paradigm (for a review see [8]). Neurophysiological evidence suggests that pairing abstract stimuli to specific actions results in Hebbian plasticity of connections between anatomically related structures that fire together (e.g. dorsal premotor cortex and putamen [9], [10]). Recently, we used abstract stimuli to study the impact of sensorimotor learning on the corticospinal excitability of adult observers [11]. Specifically, we trained subjects to learn arbitrary associations between two abstract, cues and two different actions. Subjects first learned the association between the abstract cues and the observed actions (visual symbols) through visuovisual learning and then the association between the same abstract cues and the executed actions (motor symbols) through visuomotor learning. Corticospinal excitability (CSE) was measured during passive observation of the cue and the action after each type of training. The results revealed a similar pattern of CSE for observation of new motor symbols and actions involving the recorded muscle but *not* for visual symbols, ruling out the possibility that motor facilitation reflected prediction/anticipation of the upcoming action. Our findings provide evidence supporting a role of sensorimotor learning in the development of motor resonance. Yet facilitation of motor output may not always reflect activation of the motor network. In fact, given that CSE is thought to reflect changes in membrane potential [12], TMS in combination with EMG may pick up near threshold neuronal activity of local origin that may not result in a significant change in oxygen consumption captured by indirect measures of synaptic input and/or firing rate such as fMRI.

Here, we used event-related fMRI to directly test the sensorimotor theory of motor resonance by looking for spatial overlap between the network recruited during action observation/execution and that recruited during observation of new motor symbols. Subjects learned arbitrary visuomotor associations between two colored cues and abduction finger movements (index and little finger) and another colored cue and no movement. During the scanning session, the same colored cues either preceded the observation of videos of finger movements, videos of a still hand (Observation blocks), or signaled the execution of finger movements (Action blocks). Brain activity related to the cue was distinguished from that related to execution/observation of the action for each block.

We predicted that if sensorimotor learning is at the basis of motor resonance then the network recruited by the cue when it preceded an observed action (symbolic action observation network, SAO) should overlap considerably with the network recruited during the observation and execution of finger movements (Action observation/execution network, AOE). In accordance with the sensorimotor model posited by Keysers and Perret (2004), we expected this overlap to include regions from the dorsal portion of the premotor cortex and posterior portions of the parietal cortex which are active during execution of this type of intransitive hand action rather than the classic “mirror system” (PMv/AIP). Previous work indicates that prediction of upcoming visual sequences activates a fronto-parietal network that partly overlaps with that recruited during action observation [13]. If anticipation or prediction of the upcoming stimulus, and not sensorimotor learning, was driving the functional pattern identified by the SAO then the network recruited by observation of the cue preceding a still hand (visual prediction network) should also overlap with the AOE. Finally, given that dorsal premotor and posterior parietal cortex participate in the formation and retrieval of arbitrary visuomotor associations during motor preparation [14], [15], [16] we expected the network recruited by the cue, when it preceded an executed action (motor preparation network) to partly overlap with the AOE.

## Methods

### Ethics Statement

This study was approved by the Ethics Committee of the University of Buenos Aires Hospital and carried out according to the Declaration of Helsinki. All subjects provided written informed consent.

### Subjects

Twelve healthy right-handed volunteers (5 females; between 21 and 32 years old; mean age ± SD: 27.6±5.2) participated in the study. They did not present any neurological or psychiatric disorders.

### Experimental Paradigm

#### Practice session

Subjects first learned visuomotor associations between colored cues and abduction movements of the index and little fingers before entering the scanner. On each trial, subjects were presented with a white fixation cross that became a cue when it changed color. There were three cues: the cue preceding the index finger was blue, the cue preceding the little finger was red (for practicality, we will refer to these as *dynamic cues*) and the cue preceding a static hand (no movement) was pink (we will refer to this as a *static cue*). Participants were instructed to execute the finger movement corresponding to each cue using their right hand as fast as possible after its disappearance. Accuracy and reaction time (RT) were recorded using a custom made electronic device. To avoid vision of their finger movements, subjects placed their hands in their lap and under the table, over the recording device. During training, cue duration was varied between 1000 and 1500 ms to prevent a temporal association between the cue and the movement. Trials in which subjects executed the movement before the cue ended were discarded. Ninety cues of each type were presented in a pseudorandomized order.

#### fMRI session

During the scanning session, the participants' right hand rested on a plastic slab placed to the side of the body to allow performance of the abduction movements. Subjects performed two different tasks blocks: Action (A) and Observation (O) blocks. During Action blocks subjects were required to perform either of the two symbolically instructed finger movements with the right hand upon the presentation of the dynamic cue, or to withhold any movement after the presentation of the static cue. During Observation blocks, the dynamic cues were followed by videos of a right hand shown in first person perspective performing the same finger movements (action observation condition) and the static cue was followed by a video of a still hand. Each video lasted 800 ms. Subjects were instructed to observe the videos. To ensure they paid attention to the videos, a low-contrast asterisk appeared near the moving finger at the maximal aperture in some blocks. At the end of each block, subjects were instructed to press a button (Current Designs. Inc, USA) using their left index finger, if they had seen the asterisk. To monitor movement during observation blocks, finger movements of the right hand were filmed throughout the scanning session using an optical camera placed outside the scanner room. The camera detected two markers of reflecting material placed on the tips of the index and little fingers. Instructions were repeated at the beginning of each block to remind subjects of their task.

The purpose of blocking action and observation trials was to avoid the occurrence of phenomena such as motor priming, that normally take place when perceived and executed actions are interleaved (E.g. [17]). Each A and O block consisted of 6 trials of the dynamic condition (3 index finger and 3 little finger movements) and 6 trials of the static condition. To model separately the cue and the action during Action and Observation blocks, the inter-stimulus interval was jittered semirandomly between 3 and 12 seconds (in multiples of 3). Specifically, one given ISI was repeated no more than three times in a row. Subjects underwent three fMRI runs (≅12 minutes each), except for one participant that underwent only two runs due to technical difficulties. Thus, there were a total of 36 trials per condition for each Action and for each Observation block. All stimuli were presented following a pseudo-randomized order and balanced within blocks to avoid repetitions across trials. Trials were arranged following a stochastic design with null events (variable duration of fixation between trials) [18] in 2 Action blocks (A) and 2 Observation blocks (O) per run, presented in the following order A, O, A, O.

### MRI acquisition

A unique high-resolution structural image (T1-FFE; matrix = 256×256 voxels; FOV = 256; voxel size: 1×1×1 mm, TR = 25, TE = 4.954; # slices = 160) and 248 functional echo-planar images (EPI, matrix = 64×64 voxels; FOV = 256; voxel size: 4×4×4 mm, TR = 2.901, TE = 50, # slices = 33, acquisition: descending) were acquired per run on a 1.5 T Philips Intera scanner. Three runs were acquired in total. The first 5 volumes of each run, during which stabilization of the magnetic field was achieved, were discarded for data analysis.

### Data Analysis

Data were analyzed using the Statistical Parametric Mapping software SPM5 (Wellcome Department of Cognitive Neurology, London, UK). The functional time series was motion corrected, slice timing corrected and smoothed with a Gaussian kernel of 8 mm full-width at half-maximum. Functional images were first registered to the corresponding high-resolution structural image. The latter was then transformed into the standard anatomical space [19] using the structural template of the Montreal Neurological Institute (MNI 152). These parameters were used to normalize all functional images. Following preprocessing, a high-pass filter of 128 sec was applied and serial autocorrelations were removed using an autoregressive model of first order. Two types of events were modelled per trial: one synchronized to the onset of the cue and one synchronized to the onset of the observed/executed action. The model included four regressors as effects of interest for the observation blocks and four for the action blocks: dynamic cue, action, static cue, and static hand. Each regressor was convolved with a canonical hemodynamic response function. Next, a repeated measures ANOVA was run on the 12 subjects in order to identify group effects. Finally, contrasts were coded for the conditions of interest for each block. To test for spatial overlap between conditions of interest we used a logical AND conjunction analysis, which controls for type I error (Nichols et. al., 2005). For practicality, conjunctions are indicated with the symbol ∩. Statistical inferences for contrasts of interest and conjunctions were drawn at p<0.05, corrected for multiple comparisons (Family Wise Error correction).

Based on the predictions stated in the introduction we ran the following Conjunction Analyses:

1. Action observation ∩ Action execution, (AOE network): Conjunction of Action execution network (execution of finger movements during Action blocks vs. fixation) and Action observation network (observation of finger movements during Observation blocks vs. fixation).
2. Action observation ∩ Action execution ∩ Motor preparation: Conjunction of Action execution network, Action observation network and Motor preparation network (observation of dynamic cues during Action blocks vs. fixation).
3. Action observation ∩ Action execution ∩ Symbolic action observation: Conjunction of Action execution network, Action observation network and Symbolic action observation network (observation of dynamic cues during Observation blocks vs. fixation).
4. Action observation ∩ Action execution ∩ Visual prediction: Conjunction of Action execution network, Action observation network and visual prediction network (Observation of static cues during Observation blocks vs. fixation).

## Results

### Behavioral results

The accuracy recorded during the practice session indicated that subjects performed the task correctly (correct responses followed by range for: index = 98.6% (96 to 100%), little finger = 97.2% (87.5 to 100%), static hand 99.7% (97.9 to 100)). Furthermore, the significant reduction in reaction time over training indicated that sensorimotor learning took place (first ten movements mean ± SE: 328±39 msec, last ten movements :260±29 msec; p = 0.006, paired t test). Finally, during the scanning session participants correctly detected the asterisk in all blocks (100% correct responses), suggesting they were paying attention to the stimuli.

Visual inspection of the videos for 6 out of 12 subjects obtained during the scanning session indicated that finger movements took place during Action blocks but not during Observation blocks. No data was obtained for the remaining 6 subjects due to a technical failure in analogue/digital data conversion.

### Functional MRI

**Figure 1:**
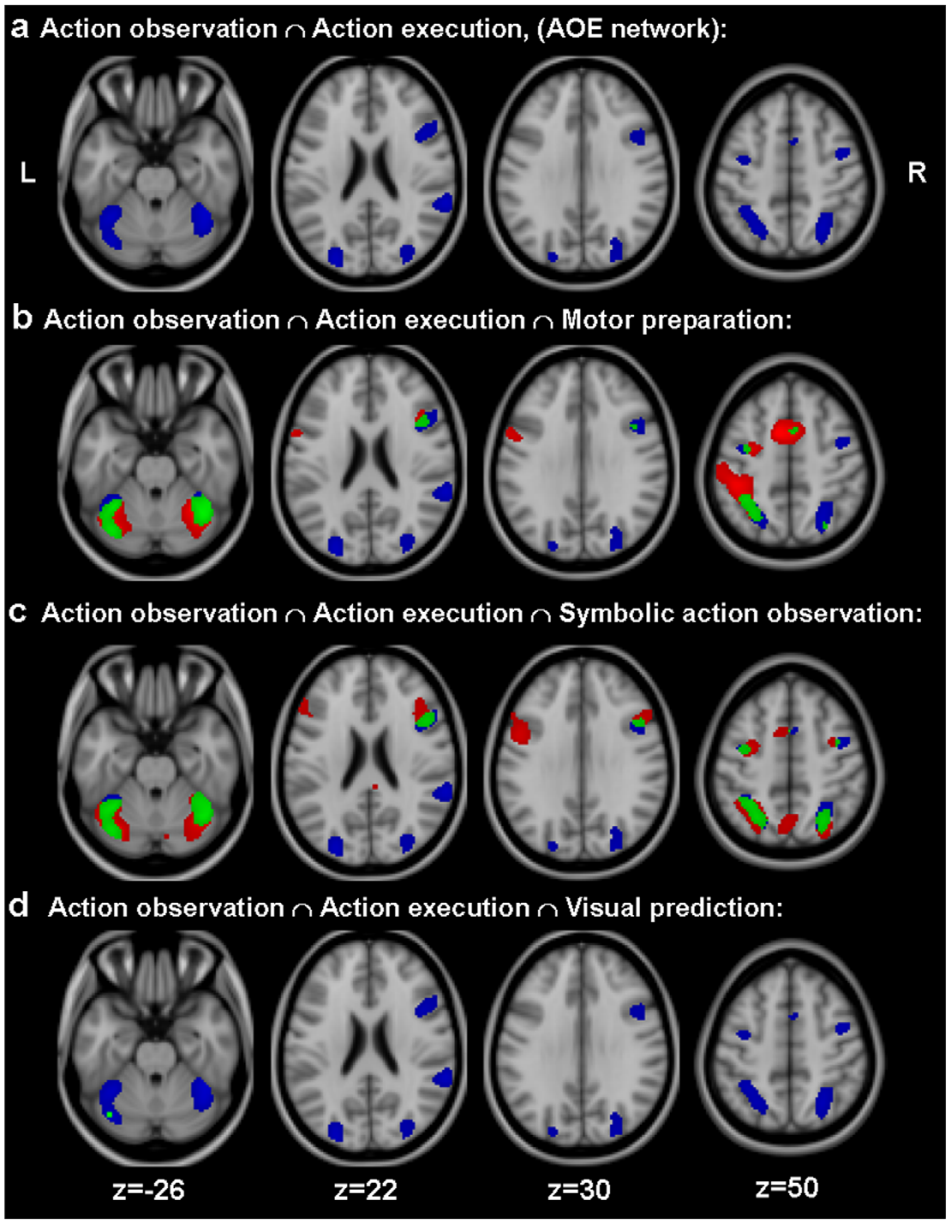
fMRI results. Statistical parametric maps (t values) for each of the four conjunction analyses described in the methods section. The SPM for AOE is depicted in blue whereas the SPM for each individual network is depicted in red. Regions in green correspond to the overlap between each individual network and the AOE identified through conjunction analysis. Statistical parametric maps for individual contrasts and conjunctions were thresholded at t>4.85 (p<0.05 corrected for multiple comparisons, FWE) and projected on the structural template of SPM (MNI 152).

Figure 1 shows the statistic parametric maps corresponding to each network and its overlap with the action observation/execution network (conjunctions). Table 1 depicts the stereotaxic coordinates (Tailarach and Tournoux, 1988) corresponding to the peak voxels for each conjunction.

**Table 1:** Stereotaxic coordinates corresponding to the four conjunctions.

Anatomical Location	MNI coordinates				
	x	y	z	t-value	p-value
**Action observation ∩ Action execution, (AOE network)**					
L middle intraparietal sulcus	−26	−64	56	7.68	<0.001
R middle intraparietal sulcus	26	−62	54	7.44	<0.001
R precentral gyrus (PMd)	44	0	52	6.45	<0.001
L precentral gyrus (PMd)	−38	−6	52	5.93	0.001
Precentral gyrus (SMA)	4	6	52	5.07	0.024
L anterior intraparietal sulcus	−34	−48	48	6.65	<0.001
R anterior intraparietal sulcus	30	−52	48	6.08	<0.001
R precentral gyrus (PMv)	52	4	38	6.14	0.001
L precentral gyrus (PMv)	−52	2	34	5.20	0.016
L lateral occipital cortex, superior division	−24	−84	28	6.08	<0.001
R lateral occipital cortex, superior division	28	−76	26	5.76	0.002
R inferior frontal gyrus (pars opercularis)	44	14	20	6.80	<0.001
R superior temporal gyrus/supramarginal gyrus	62	−40	20	8.02	<0.001
L cerebellum (HV/HVI)	−34	−54	−26	7.40	<0.001
R cerebellum (HV/HVI)	34	−52	−26	7.68	<0.001
**Action observation ∩ Action execution ∩ Motor preparation**					
R middle intraparietal sulcus	28	−68	56	5.70	0.003
L middle intraparietal sulcus	−30	−60	54	6.87	<0.001
L precentral gyrus (PMd)	−36	−8	54	5.79	0.002
Precentral gyrus (SMA)	4	6	52	5.07	0.024
L anterior intraparietal sulcus	−34	−48	48	6.61	<0.001
L postcentral gyrus*	−42	−34	48	8.22	<0.001
R precentral gyrus (PMv)	50	2	34	4.91	0.041
L precentral gyrus (PMv)*	−52	2	34	5.20	0.016
R inferior frontal gyrus (pars opercularis)	40	12	26	5.77	0.002
R superior temporal gyrus/supramarginal gyrus	60	−40	14	5.48	0.006
L cerebellum (HV/HVI)	−34	−54	−26	7.40	<0.001
R cerebellum (HV/HVI)	38	−58	−26	7.98	<0.001
**Action observation ∩Action execution ∩ Symbolic action observation**					
Precuneus*	−2	−56	58	5.55	0.005
R middle intraparietal sulcus	28	−64	56	6.90	<0.001
L middle intraparietal sulcus	−28	−62	52	6.77	<0.001
L precentral gyrus (PMd)	−36	−8	52	5.81	0.002
Precentral gyrus (SMA)	0	8	50	4.86	0.049
L anterior intraparietal sulcus	−34	−48	48	6.60	<0.001
R precentral gyrus (PMd)	38	−2	48	5.21	0.015
Precuneus*	2	−66	44	5.94	0.001
L precentral gyrus (PMv)*	−52	2	34	5.20	0.016
L inferior frontal gyrus (pars opercularis)*	−54	20	26	5.23	0.013
R inferior frontal gyrus (pars opercularis)	46	16	24	6.08	0.001
R superior temporal gyrus/supramarginal gyrus	62	−40	18	5.28	0.012
R cerebellum (HV/HVI)	34	−62	−24	7.49	<0.001
L cerebellum (HV/HVI)	−34	−54	−26	7.36	<0.001
**Action observation ∩ Action execution ∩ Visual prediction**					
L cerebellum (HV/HVI)	−36	−54	−28	6.540	<0.001

Shown are the stereotaxic coordinates (Talairach and Tournoux (1988), MNI 152 template) for peak voxels, t values (20 conditions in total, having 19 degrees of freedom, leave 77 degrees of freedom from 96 images) and p values for the four conjunctions depicted in Figure 1. Asterisks indicate those regions that were part of the corresponding network but did not appear significant in the conjunction analysis. All *p* values are corrected for multiple comparisons (Family Wise Error).

### - Action observation ∩ Action execution, (AOE network)

The functional network recruited during both observation and execution of finger movements included bilateral dorsal premotor cortex (PMd), bilateral anterior and middle portions of the intraparietal sulcus (IPS), supplementary motor area, right inferior frontal gyrus (pars opercularis), right supramarginal gyrus/superior temporal gyrus, right ventral premotor cortex (PMv) and bilateral anterior cerebellum (lobules V/VI) (Figure 1a, in blue).

### - Action observation ∩ Action execution ∩ Motor preparation

Observation of dynamic cues during Action blocks activated the anterior portion of the left dorsal premotor cortex, the left ventral premotor cortex, the left somatosensory cortex, the anterior and middle cortex in the vicinity of the left intraparietal sulcus, the supplementary motor area, the right inferior frontal gyrus and the bilateral cerebellum (lobules V/VI). Part of the right ventral premotor cortex and the middle portion of the posterior parietal cortex were also recruited in this condition but to a lesser extent (Figure 1.b, in red). The conjunction analysis detected significant overlap with the AOE at the level of the SMA, left dorsal premotor cortex, left posterior parietal cortex, right inferior frontal gyrus and bilateral cerebellum (Figure 1.b, in green). Small overlap was observed over the right superior parietal lobule and left ventral premotor cortex.

### - Action observation ∩ Action execution ∩ Symbolic action observation

Observation of dynamic cues during Observation blocks activated a functional pattern of brain activity that included bilateral dorsal premotor cortex, bilateral anterior and middle portions of the IPS, supplementary motor area, bilateral inferior frontal gyrus (pars opercularis), left ventral premotor cortex, bilateral anterior cerebellum (lobules V/VI) and the precuneus (Figure 1c, red). The conjunction analysis indicated that, with exception of the left ventral premotor, left inferior frontal gyrus and the precuneus, all these areas overlapped with those recruited during AOE (Figure 1c, in green).

### - Action observation ∩ Action execution ∩ Visual prediction/anticipation

Only a small region in the left cerebellar hemisphere was detected for the visual prediction network. This region showed a small overlap with the AOE as indicated by the conjunction analysis (Figure 1.d, in green).

## Discussion

The neural mechanisms at the basis of motor resonance are of major interest to the field of motor control. Here, we aimed to directly test the sensorimotor learning theory of motor resonance by creating new visuomotor representations using abstract stimuli (motor symbols), and identifying the network associated with them through functional magnetic resonance imaging. We predicted that, if sensorimotor learning was at the basis of motor resonance, then the network recruited during observation of new motor symbols should considerably overlap with that recruited through observation/execution of the original action.

Action observation/execution of abduction finger movements recruited a network including the bilateral anterior portion of the dorsal premotor cortex, bilateral posterior parietal cortex (anterior and middle portions of the IPS), supplementary motor area, right ventral premotor, inferior parietal cortex and bilateral cerebellum. This functional network is not consistent with the classic “mirror system” localized to PMv and AIP [1], [2]. Our results are in line with other imaging studies using intransitive hand movements [20], [21], [22], [23] and with a TMS study showing facilitation of corticospinal excitability during observation of finger movements through pre-pulse conditioning of both dorsal and ventral premotor cortex [24]. The PMv/IFG has been long postulated to be the human homologue of F5, where neurons with mirror properties were first characterized, and to code for hand/object interactions and/or action goals [25], [26], [27]. The fact that, in our study, these regions were active through observation of intransitive finger movements that, by definition, lack a goal is inconsistent with this view and rather suggests that the ventral motor cortex may deal with more basic aspects of the stimulus, the perspective from which actions are perceived [28] or the stimulus-movement association, such as the identification of the effector [29].

Observation of new motor symbols recruited a very similar network which included the bilateral precuneus. In addition, the IFG and PMv were activated bilaterally. The tight overlap between the SAO network and the AOE network is consistent with our prediction and supports the hypothesis that sensorimotor learning is at the basis of motor resonance. One could claim that the SAO network may not support motor resonance but the prediction/anticipation of an upcoming visual stimulus [13], [29]. In order to address this hypothesis we identified the network recruited by observation of an abstract cue anticipating a video of a static hand and examined its overlap with the AOE network. Only a small region of the left cerebellar cortex overlapped with the AOE network, suggesting that visual prediction/anticipation was unlikely to be driving the functional pattern identified during observation of new symbols.

In contrast, the brain regions activated during motor preparation overlapped substantially with the AOE network. These included mostly the SMA, the anterior portion of the left PMd, the cortex in the vicinity of the left middle IPS and the right inferior frontal gyrus. Lesions of the anterior portion of the dorsal premotor cortex prevents learning and retrieval of arbitrary visuomotor associations between abstract stimuli and actions involving finger movements or button presses [15], [30]. Neuroimaging studies further reveal that SMA and the cortex in the vicinity of the IPS are typically activated once performance has reached a plateau, suggesting they may be part of the retrieval network [16], [31], [32], [33], [34]. Passingham has hypothesized that, after learning, the abstract cue elicits the recall of the action appropriate for the context through PMd [35]. In this view, the role of PMd would be analogous to that assigned to neurons of PMv during observation of actions involving grasping. The overlap found in our study between the SAO, the motor preparation and the AOE network, provides new evidence extending Passingham's statement to motor resonance. The left lateralization of the functional pattern for fronto-parietal regions is likely to reflect the concomitant preparation for the execution of the upcoming action.

We have recently demonstrated that viewing abstract stimuli previously paired with an abduction finger movement induces a similar pattern of corticospinal excitability in the observer to the one elicited during direct observation of the corresponding finger movement [11]. Our fMRI study complements and strengthens our previous work. The lack of activation of the primary motor cortex for the SAO network suggests that CSE facilitation was unlikely the result of a local process. Instead, the wide overlap between the AOE and the SAO networks suggests that changes in CSE that emerged through sensorimotor learning were likely the result of a widespread network upstream to the motor cortex. Previous attempts have been made at testing the sensorimotor theory of motor resonance indirectly, by manipulating the congruency of training (Catmur et. al., 2008). fMRI has shown that incongruent sensorimotor training (e.g. moving a foot while observing a hand movement and viceversa) modulates the activity of the action observation execution network recruited after congruent training (e.g. moving a foot while observing a foot) [7]. However, given that incongruent training involves the reconfiguration of an existing motor representation, it is likely that the reported modulation of the action/observation network partly reflected inhibitory processes (Catmur et. al., 2008). The use of new symbols in our study ruled out inhibition as a confounding factor and allowed to directly assess the effect of sensorimotor learning on motor resonance.

In addition, we believe that the use of abstract symbols shed new light on some unresolved issues regarding the relevance of the observed stimulus for action observation. Converging experimental evidence indicates that the dynamic component of an action is not necessary to activate the motor system of the observer. Indeed, viewing static pictures of hands implying action [36], pictures of hand-object interactions [25] or 3D objects [37] is sufficient to activate the motor system in the observer. Grafton and collaborators (Grafton et. al., 1997) have previously hypothesized that the sole observation of a stimulus that has been repeatedly associated with an action through conditional motor learning (e.g. a tool) is sufficient to retrieve the corresponding motor representation. The results of our previous and current study, based on newly learned symbols, are consistent with Grafton's hypothesis and further indicate that the physical implication of action is not a requisite to elicit motor resonance in the observer; what seems to matter is the sensorimotor representation.

Despite its strengths, there were certain limitations to our study that need to be addressed. The scanner used was a 1.5 Tesla and the total number of subjects was relatively small (12 subjects). This may have precluded the detection of changes in synaptic input to primary motor cortex. M1 activation has been detected during action observation with electrophysiological techniques (Tkach et al., 2007) and with fMRI [38]. Another limitation of our study was the lack of a sensitive measure of muscle activity in the scanner. Although no overt movements were detected in half of the subjects through a videocamera, electromyographic measurement would have definitively ruled out muscle activity during Observation blocks. From a functional point of view, it is important to acknowledge that a conjunction analysis does not allow discerning whether the AOE and the symbolic action observation network carry out the same neural computations. Although this question posits a challenge at the low spatial spatio-temporal resolution of fMRI, the use of a repetition suppression protocol could provide further insight into the common synaptic pathways at the basis of motor resonance and sensorimotor learning. Finally, unlike a large body of evidence supporting bilateral activation of the inferior frontal and ventral premotor cortex during observation of actions involving hand/object interactions [39], the spatial pattern elicited by the AOE network was right lateralized. This included a region between the superior temporal gyrus (STG) and supramarginal gyrus (SMG). It has been suggested that viewing meaningless human motion elicits relatively more activation of the right hemisphere than viewing meaningful human actions [23]. In contrast, a recent study by Newman-Norlund and collaborators [40] reported a different functional pattern linking right SMG with the processing of meaningful actions and left SMG with the processing of meaningless actions. It is important to notice, however, that meaningless actions were not intransitive movements, as used in more studies, but actions violating the congruency between the effector and the object acted upon (e.g. stapler operated by foot instead of hand). This type of higher-order processing requiring access to semantic information may likely depend on left hemisphere processing. Based on these and our findings it is possible that actions deprived of semantic meaning such as finger movements are processed in terms of their physical (object recognition) and spatial features by the right hemisphere whereas actions involving more complex object interactions such as tool use, may require bilateral processing. This interpretation is consistent with the observation that, unlike the AOE network, the SAO network associated with the retrieval of motor symbols activated IFG and PMv bilaterally.

There is little controversy that the motor system can be activated by passive viewing of movement or actions. Growing experimental evidence, however, including our current findings, are not consistent with the traditional view that motor resonance originates from a “mirror system” restricted to neurons in PMv and AIP. Neurophysiological studies indicate that both dorsal premotor and primary motor neurons show increased firing rates when macaques observe reaching movements to visual targets [41], [42]. Neurons with mirror characteristics have also been reported in other regions of the parietal cortex such as the lateral intraparietal area [43] and the ventral intraparietal area [44] during the perception of gaze and movements of body parts, respectively. Furthermore, single-unit recordings in humans indicate that viewing grasping actions and facial expressions is associated with greater activity in neurons of the supplementary motor area, hippocampus, parahippocampal gyrus and entorhinal cortex [45]. These findings are consistent with one of two hypotheses: i) motor resonance emerges from neurons with special mirror properties widespread throughout the brain, or ii) motor resonance may simply reflect the reactivation of specific motor programs. The former view would be more congruent with a distributed than with a systems framework of brain function. The latter view, on the other hand, would not require the existence of specialized neurons as it would be based on the association between coactive sensory and motor regions. This mechanism presents a potential advantage from an evolutionary viewpoint in that it would rely on neuronal circuits supporting motor control, learning and memory. In this view, motor resonance could simply be explained by the retrieval of the motor program associated with the observed action and its covert activation. Our finding that viewing a motor symbol retrieves the motor network recruited during training is consistent with the latter view.
